# Standardized Uptake Values on SPECT/CT: A Promising Alternative Tool for Treatment Evaluation and Prognosis of Metastatic Neuroendocrine Tumours

**DOI:** 10.3390/diagnostics13020318

**Published:** 2023-01-15

**Authors:** Mirela Gherghe, Alexandra Maria Lazar, Laurentiu Simion, Ionela-Nicoleta Irimescu, Maria-Carla Sterea, Mario-Demian Mutuleanu, Rodica Maricela Anghel

**Affiliations:** 1 Nuclear Medicine Department, University of Medicine and Pharmacy “Carol Davila” Bucharest, 050474 Bucharest, Romania; 2 Nuclear Medicine Department, Institute of Oncology “Professor Doctor Alexandru Trestioreanu”, 022328 Bucharest, Romania; 3 Department of Surgery, “Carol Davila” University of Medicine and Pharmacy, 050474 Bucharest, Romania; 4 1st Clinic of General Surgery and Surgical Oncology, Bucharest Oncology Institute, 022328 Bucharest, Romania; 5 Oncology Department, University of Medicine and Pharmacy “Carol Davila” Bucharest, 050474 Bucharest, Romania; 6 Radiotherapy Department, Institute of Oncology “Professor Doctor Alexandru Trestioreanu”, 022328 Bucharest, Romania

**Keywords:** ^99m^TcEDDA/HYNIC-TOC, neuroendocrine tumors, quantitative SPECT/CT, SPECT/CT, ^68^Ga-DOTA-PET/CT, metastatic disease, somatostatin receptors

## Abstract

(1) Background: The aim of our study was to assess the feasibility of ^99m^TcEDDA/HYNIC-TOC SPECT/CT quantitative analysis in evaluating treatment response and disease progression in patients with NETs. (2) Methods: This prospective monocentric study evaluated 35 SPECT/CT examinations performed on 14 patients with neuroendocrine tumours who underwent a baseline and at least one follow-up ^99m^TcEDDA/HYNIC-TOC scan as part of their clinical management. The examination protocol included a whole-body scan acquired 2 h after the radiotracer’s administration, with the SPECT/CT performed 4 h post-injection. Images were analyzed by two experienced physicians and patients were classified into response categories based on their changes in SUV values. (3) Results: We evaluated 14 baseline studies and 21 follow-up scans, accounting for 123 lesions. A statistically positive correlation has been found between the SUV_max_ and SUV_peak_ values in tumoral lesions (*p* < 0.05). No correlation has been found between the SUV values and the ki67 proliferation index. Finally, 64.29% patients were classified as SD at the end of the study, with only 14.29% of patients exhibiting PD and 21.43% patients with PR. (4) Conclusions: The quantitative analysis of ^99m^TcEDDA/HYNIC-TOC SPECT/CT data in patients with neuroendocrine tumours could represent an alternative to ^68^Ga-DOTA-peptides PET/CT for the monitoring and prognosis of NETs.

## 1. Introduction

Neuroendocrine neoplasms (NENs) are a group of heterogenous malignancies that originate from diffuse neuroendocrine cells dispersed throughout the body which possess secretory properties which lead to their specific syndromes of uncontrolled hormone hypersecretion [1,2]. NENs have been described in the nervous system, respiratory tract, gastrointestinal tract, thyroid, breast, skin and urogenital system, most commonly the gastro-entero-pancreatic (60% of the cases) and pulmonary (30% cases) [2,3]. Overall, considered as indolent tumours, their prognosis varies by morphology, proliferation rate, primary tumoral site and stage at diagnosis [3].

The World Health Organization (WHO) introduced a new classification of the NENs in 2019, stating a clear distinction between well-differentiated neuroendocrine tumours (NETs), also known as carcinoid tumours, and poorly differentiated neuroendocrine carcinomas (NECs) [4,5]. The latest update in the 2022 WHO classification of neuroendocrine tumours consolidates the utility of this dichotomous morphological subdivision made on criteria based on the tumoral differentiation: NETs are generally (but not always) graded as G1, G2 or G3, usually presenting a low ki67 index, while NECs are by definition high grade (ki67 > 55%), presenting cellular polymorphism [6]. NETs may display as functionally active or non-functional tumours, presenting distinct clinical characteristics based on their site of origin and having specific features that can represent a target for molecular imaging and peptide receptor radionuclide therapy (PRRT), such as expression somatostatin receptors (SSTRs), especially subtypes 2 and 5 [6,7,8,9].

Functional molecular imaging of NETs plays a crucial role in initial tumoral staging, disease assessment after therapy (restaging), and follow-up and planning for PRRT [10]. Through the years, many radiotracers have been developed for human use, with dissimilar sensitivity, specificity and diagnostic accuracy, as they differ in terms of radionuclides, chelators and affinity for SSTR subtypes [11]. Although for a long time somatostatin receptor scintigraphy (SRS) with ^111^In-DTPA-DPhe^1^-octreotide [^111^In-pentetreotide] (Octreoscan^®^) has been considered the “gold standard” for diagnosis and monitoring, recently developed radiopharmaceuticals, labeled with ^99m^Tc and ^68^Ga, which present a higher sensitivity for tumour detection and a lower mean effective dose for the patient, have added to the enhancement in the resolution of the new single-photon emission computed tomography (SPECT) and positron emission tomography (PET) scanners, and have limited the use of ^111^In-pentetreotide SRS and favoured the use of these new radiotracers [6,12,13]. ^99m^Tc-labelled peptides have been introduced to the practical clinic recently, and demonstrated some advantages compared with ^111^In-pentetreotide, with better imaging quality on gamma cameras and a lower radiation burden for patients which allows the usage of higher radiotracer doses, making them an alternative to ^68^Ga-DOTA-PET examinations in centres that do not benefit from PET-scanners or ^68^Ge/^68^Ga generators [14,15,16,17,18,19,20,21]. A late review by Poletto et al. [20] made a tight comparison between SPECT and PET radiopharmaceuticals, highlighting the better specificity and sensitivity in diagnosis and monitoring of NETs of the latter, but still underlining the higher sensitivity of 99mTc-based SPECT radiotracers compared to the ^111^In-labelled ones. In a recent paper, we also demonstrated the good diagnostic performances of 99mTc-based radiopharmaceutical 99mTcEDDA/HYNIC-TOC, which has a high affinity for SSTR2 and a lower one for SSTR3 and SSTR5, in the management of patients suffering from gastro-entero-pancreatic NETs, obtaining results that make it a good alternative to ^68^Ga-DOTA-peptides [21].

Furthermore, advances in the development of SPECT cameras that raised the opportunity for dosimetry and uptake quantification can make ^99m^TcEDDA/HYNIC-TOC SPECT/CT suitable for directing PRRT with beta or alpha radiation-releasing radiolabeled somatostatin analogues, allowing for the monitoring of treatment response and patient follow-up [22]. The introduction of hybrid devices, combining SPECT gamma cameras with a computed tomography (CT) scanner, enabled the quantitative evaluation of images through new techniques of image reconstruction, reducing errors in absolute quantification in SPECT by performing scatter and attenuation corrections [23].

The purpose of our study is to assess the feasibility of quantitative ^99m^TcEDDA/HYNIC-TOC SPECT/CT in evaluating treatment response in patients with NETs, with the aim of providing an alternative to PET/CT with ^68^Ga-labeled peptides.

## 2. Materials and Methods

### 2.1. Patient Selection

Our study included prospective data from patients enrolled after fully understanding and agreeing to the institutional informed consent. The study was conducted in the Nuclear Medicine Department of the Institute of Oncology “Prof. Dr. Alexandru Trestioreanu”, Bucharest, Romania, from November 2019 to October 2022. The reference population was represented by patients diagnosed with neuroendocrine tumours who underwent at least two consecutive ^99m^TcEDDA/HYNIC-TOC SRS in our centre.

Immunohistochemical tumoral markers: somatostatin receptors (SSTR), Chromogranin A (CGA), Synaptophysin (SYN), CD56, CDX2, proliferation index ki67, Neuron Specific Enolase (NSE), seric biochemical markers: Chromogranin A (CGA), serotonin, urinary 5-hydroxyindoleacetic acid (5-HIAA), and information on the treatment protocols used during the follow-up period were collected from the medical records of the patients.

The inclusion criteria for the study were the following: (1) a histopathologically confirmed neuroendocrine tumour (either from biopsy or surgical procedures), with clearly established values for the ki67 proliferation index; (2) at least two lesions presenting the uptake of ^99m^TcEDDA/HYNIC-TOC on the baseline SPECT/CT scan; (3) at least two consecutive SPECT/CT scans within a 4-12 month timeframe; (4) information on the acquisition protocol for both baseline and follow-up scans (measured prepared activity, time of measurement, injection time and residual syringe activity).

All patients were following treatment with long-acting release somatostatin analogues (SSA), either octreotide at a dosage of 20–40 mg or lanreotide in a dosage of 60–120 mg, both were administered subcutaneously at an interval of 28 days. Patients were requested to interrupt their administration at least 3–4 weeks prior to the investigation.

Considering the selection criteria and the relatively low incidence of these types of tumours, 14 patients eventually included in our study.

### 2.2. ^99m^Tc-EDDA/HYNIC-TOC Synthesis

The peptide HYNIC-[D-Phe1Tyr3-Octreotide] trifluoroacetate was provided by IAE POLATOM (Otwock, Poland) in the commercial Tektrotyd^®^ kit. The preparation of ^99m^Tc-EDDA/HYNIC-TOC was performed according to the manufacturer’s instructions, consisting of radiolabeling of HYNIC-[D-Phe1Tyr3-Octreotide] trifluoroacetate with ^99m^Tc in a preheated reaction vial at 80 °C, just prior to intravenous administration.

### 2.3. ^99m^Tc-EDDA/HYNIC-TOC Radiochemical Purity

The radiochemical purity of the ^99m^Tc-EDDA/HYNIC-TOC was checked using radio thin-layer chromatography (radio-TLC) (POLATOM, Otwock, Poland) before each patient administration, as radio-TLC is preferred over radio-high-performance liquid chromatography due to its simplicity and rapidity [24]. The radiopharmaceutical was administered only if its measured radiochemical purity was higher than 95%.

### 2.4. Acquisition Technique and Reconstruction

Patient preparation included increased hydration, light food intake and mild laxatives at least 24 h before the examination.

For each scan, patients received an intravenous dose of 628–740 MBq (17–20 mCi) of ^99m^Tc-EDDA/HYNIC-TOC. The examination protocol included a “whole-body” (WB) scan performed 2 h after the radiotracer’s administration and a SPECT/CT exam 4 h post-injection. The gamma camera used for the exams was a dual-head Discovery 670 DR SPECT/CT system (General Electric Healthcare, Chicago, Illinois, United States of America) with low energy high resolution (LEHR) collimators. The planar scan was acquired using the following parameters: a pallet speed of 10 cm/min, a 128 × 128 matrix size, and a ^99m^Tc energy window. SPECT/CT imaging (thoracic, abdominal and pelvic regions) was executed using a standard protocol: a 128 × 128 matrix size, a step and shoot rotation time of 30s/view (for a total of 120 views), and a dual energy window (140 keV ± 10% and 120 keV ± 5%). After the SPECT acquisition, a low-dose CT scan was performed maintaining the patient in the same position, at 120 keV, 40–340 mA. CT images were acquired using a standard 3.75 slice thickness, then reconstructed using the standard (slice thickness of 2.5 mm), bone plus (1.25 mm) and lung (1.25 mm) reconstruction filters provided by the vendor.

The acquired SPECT/CT data were reconstructed using the Q.Volumetrix software provided by the manufacturer, using the ordered subset expectation maximization (OSEM) iterative reconstruction algorithm (8 subsets and 10 iterations), with attenuation correction, scatter correction and resolution recovery performed.

### 2.5. Scanner Calibration

Scanner calibration was performed to allow better and more precise measurements of ^99m^TcEDDA/HYNIC-TOC uptake in the targeted lesions. The SPECT/CT scanner was calibrated by scanning a uniform Jaszczak phantom without any inserts inside and a NEMA phantom with hot inserts, calculating the planar and SPECT sensitivity. The conversion factor for the sensitivity calculated between the two aquisitions showed a deviation in the range of 0-5%. According to GE Healthcare, accuracy is calculated based on the following equation:

$$Accuracy\left(\%\right)=\left(1-\frac{\left|A_{c,sph,j-a_{c,sph,j}}\right|}{A_{c,sph,j}}\right)\times 100$$

where $A_{c,sph,j}$ is the true activity concentration in the spheres and $a_{c,shp,j}$ is the activity concentration measured in the reconstructed spheres.

The measurement accuracy for 10 iterations and 8 subsets was higher than the 90% value recommended by the camera vendor (General Electric Healthcare).

### 2.6. Image Analysis

The images were interpreted independently by two experienced physicians on a dedicated workstation for imaging diagnosis (GE Xeleris V, General Electric Healthcare). Normal physiological ^99m^Tc-EDDA/HYNIC-TOC uptake was visually established in organs, using the CT images and clinical correlations. Pathological uptake was assessed in the primary lesions and the sites of the metastases. A quantitative analysis was performed in the reconstructed images using the software tool provided by the camera’s vendor (GE Q.Volumetrix, GE Xeleris V, General Electric Healthcare). The delineation of the volumes of interest (VOI) were drawn semiautomatically, and SUV_max_ and SUV_peak_ based on lean body mass (SUV_lbm_) were the elected methods in calculating the radiotracer’s uptake. SUV_max_ values for normal tissue (liver, spleen and bone) were obtained by positioning a sphere of 10 mm radius inside imagistically normal tissue. We have chosen to utilise SUV_lbm_ as it has long been recommended in practical use because it excludes the percentage of a patient’s body fat from its equation, which would raise the possibility of SUV calculation errors [25].

Quantitative analysis statistics are calculated using the following formulas [26]:

$$\mathrm{SUVlbm}\;=(\mathrm{SPECT}\;\mathrm{image}\;\mathrm{Pixels}\;\mathrm{uptake}\;(\mathrm{Bqml}))\;\times \;\frac{\left(LBM\;in\;kg\right)}{\left(actual\;activity\right)\cdot 1000}\mathrm{units}\;g/\mathrm{mL}$$

$$\mathrm{For}\;\mathrm{Males}:\;\mathrm{LBM}\;\mathrm{in}\;\mathrm{kg}=1.10\;\times \;(\mathrm{weight}\;\mathrm{in}\;\mathrm{kg})-120\;\times \;[\frac{\left(weight\;in\;kg\right)}{\left(height\;in\;cm\right)}{]}^{2}$$

$$\mathrm{For}\;\mathrm{Females}:\;\mathrm{LBM}\;\mathrm{in}\;\mathrm{kg}=1.07\;\times \;(\mathrm{weight}\;\mathrm{in}\;\mathrm{kg})-148\;\times \;[\frac{\left(weight\;in\;kg\right)}{\left(height\;in\;cm\right)}{]}^{2}$$

Actual activity = decay scan × decay1 × (Measure Activity − (decay2 × post injection activity))

Λ = 0.693/half Lifetime

decay1 = exp (λ × (Measured Time − Administered Time))

decay2 = exp (λ × (postinjection Time − Measured Time))

decay scan (injection-scan time) = exp (λ × (administered Time-scan Time))

LBM in kg = calculated using the dedicated formula for each gender

Measure Activity = preinjection activity

Measured Time = preinjection time

As precise SPECT/CT criteria for the characterization of treatment response have yet to be developed, we decided to adapt the PERCIST 1.0 criteria for our study [27]. Thus, treatment efficacy or disease progression were evaluated by comparing the summed SUV_max_ values obtained from up to five lesions between the baseline and the follow-up examinations, the results being classified as: complete response (CR)—no uptake in any of the baseline lesions and no new lesions on the follow-up study; partial response (PR)—a decrease >30% in the summed SUV_max_ value on the follow-up study; stable disease (SD)—a decrease or increase in the summed SUV_max_ value <30%; and progressive disease (PD)—new tumoral foci or an increase >30% in the summed SUV_max_ value on the follow-up study. For a better assessment of our results, we used radiology reports (either contrast-enhanced CT or magnetic resonance imaging) as a standard of reference for each patient, in correlation to both the baseline and the follow-up scans.

### 2.7. Statistical Analysis

Data analysis was performed using IBM SPSS Statistics Version 26 (IBM, SPSS, Inc., Chicago, IL, USA, 2019). Data were expressed as mean ± standard deviation (SD) and percentages. A paired samples T-test was used to compare the results. Correlations between ki67 and SUV values, as well as between SUV_max_ and SUV_peak_, were made using a Pearson correlation test. A *p* value < 0.05 was considered as statistically significant.

## 3. Results

We evaluated 35 studies (14 baseline scans and 21 follow-up scans) from 14 patients with a mean age 48.36 ± 16.93 years old (range 18–69) who had undergone a neuroendocrine scintigraphy with SPECT/CT in our clinic. Most of our patients were males (64.3%), while the most frequent neuroendocrine tumour type was pancreatic NET (42.9%), followed by NETs of the small bowel (21.4%) and pulmonary NETs (21.4%). All of our patients had metastatic disease at the inclusion in the study, the most frequent site for metastases being the liver (n = 11, 78.6%), followed closely by the lymphatic system (n = 10, 71.4%). The main patient characteristics are presented in Table 1.

All patients underwent treatment with long-acting somatostatin analogues between the baseline and the follow-up scan. The mean number of treatment cycles received by our patients between two consecutive SPECT scans was 7.76 ± 3.08 (range 3–11 cycles).

All of our patients had well differentiated tumours (NET G1—42.9%, NET G2—50%), with the exception of one, who presented a poorly differentiated NET (G3), with a ki67 index of 25%. The mean ki67 proliferation index in our patient group was 7 ± 6.25. The main neuroendocrine immunohistochemical and biochemical markers are presented in Table 2.

A total of 123 lesions were analysed, out of which 64 liver metastases (52%), 25 metastatic lymph nodes (20.3%), 7 bone metastases (5.7%) and 27 (22%) were lesions with other origins (primary tumours, peritoneal metastases, pleural metastases). SUV_max_ and SUV_peak_ were extracted for each lesion.

We performed a separate analysis regarding the ^99m^Tc-EDDA/HYNIC-TOC uptake in the most frequent metastatic sites, comparing them to the uptake normal tissue (Table 3). Mean SUV_max_ values were higher in liver and lymph nodes metastases (12.44 ± 7.76 g/mL, respectively 11.98 ± 10.45 g/mL) compared to the bone lesions, which presented a mean SUV_max_ of 5.90 ± 3.68 g/mL, probably correlated to the lower SSTR expression in osseous tissue.

SSTR-expressing malignant and non-malignant tissues showed statistically significant differences for all three types of the analysed metastases: SUV_max_ in metastases were higher than SUV_max_ in health tissue (*p* < 0.05 for all). Target lesion to normal tissue (TL/N) ratios of liver and bone metastases were generally high: 4.20 ± 2.67 for liver lesions and 9.37 ± 2.91 for bone lesions (with normal vertebrae as healthy tissue). The TL/N ratio for lymph metastases was relatively low compared to the other two (1.84 ± 1.53), as spleen was used as a reference for normal tissue and the physiological uptake of ^99m^Tc-EDDA/HYNIC-TOC is relatively high (the mean SUV_max_ in spleen was 8.01 ± 5.31). The calculation of TL/N ratios in lymph node metastases was done in nine patients, as one of them underwent a splenectomy as part of their surgical treatment.

The mean SUV_peak_ was in accordance with the SUV_max_ values, observing higher SUV_peak_ values in hepatic and lymphatic lesions (10.15 ± 6.79 g/mL, respectively 8.81 ± 7.99 g/mL), than in bone metastases (5.55 ± 2.47 g/mL) (Table 4).

**Figure 1:**
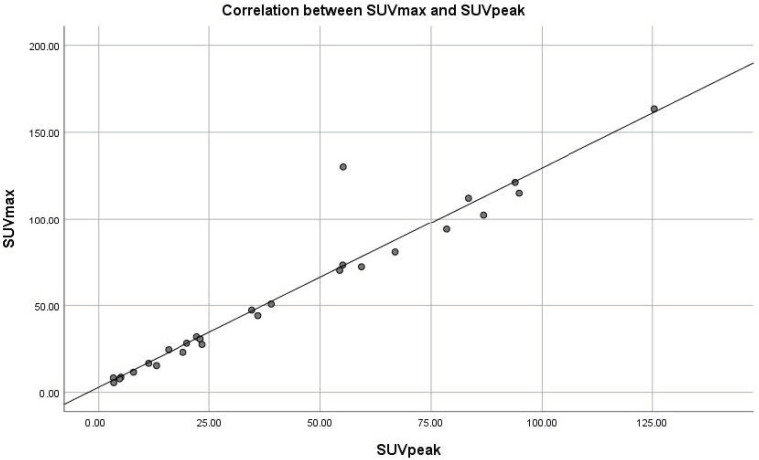
Correlation between SUV_max_ and SUV_peak_ values in NET lesions.

SUV_max_ and SUV_peak_ of the three metastatic sites showed a statistically significant positive correlation (*p* < 0.05 in all three cases), meaning that SUV_peak_ increases similarly to the elevation of SUV_max_ in NET lesions (Figure 1).

A correlation analysis between the SUV_max_ and SUV_peak_ and the ki67 proliferation index was performed in order to determine whether ^99m^Tc-EDDA/HYNIC-TOC uptake varies with the tumoral differentiation grade, but our results invalidated this hypothesis, as no statistically significant correlation was found between the three parameters. The results can be observed in Table 5.

**Figure 2:**
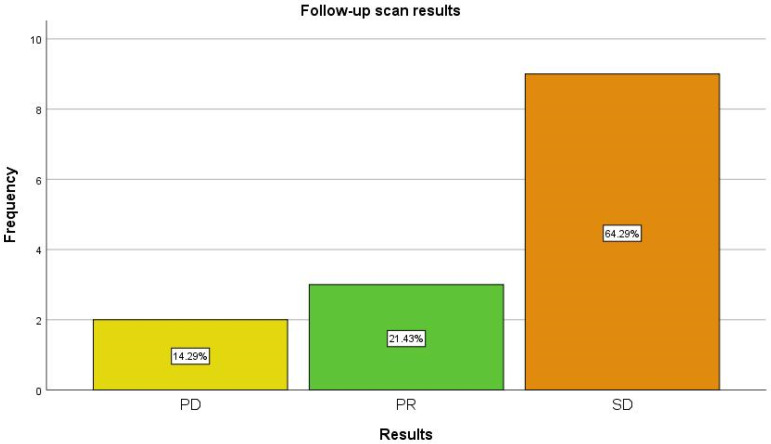
Patient distribution after follow-up examinations. Abbreviations: PD—progressive disease; PR—partial response; SD—stable disease.

For the assessment of the utility of quantification of SPECT/CT data in monitoring the patients diagnosed with NETs, we evaluated a baseline scan and at least one follow-up scan for each patient, resulting in a total of 35 examinations that were finally included in our study. The mean clinical follow-up period was 8.76 ± 3.08 months (range 4–12 months). Four of our patients had more than just one follow-up scan, at the recommendation of their clinician; however, for their inclusion in a response category, only the conclusion of the last performed scan was counted. The patient distribution after follow-up evaluation is shown in Figure 2.

**Figure 3:**
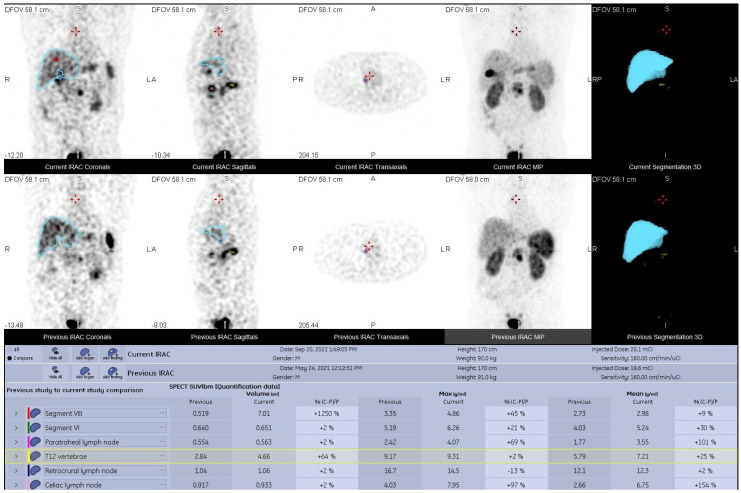
Comparison between a baseline and a follow−up scan of a 56−year−old male patient, diagnosed with an intestinal NET, grade G1, with hepatic, lymphatic and osseous metastases. The patient had been following treatment with long acting SSA. Although the liver metastasis and the paratraheal and celiac lymph nodes presented a notable increase in ^99m^Tc−EDDA/HYNIC−TOC uptake, the final assessment of the follow-up study established that the patient had stable disease (SD).

**Figure 4:**
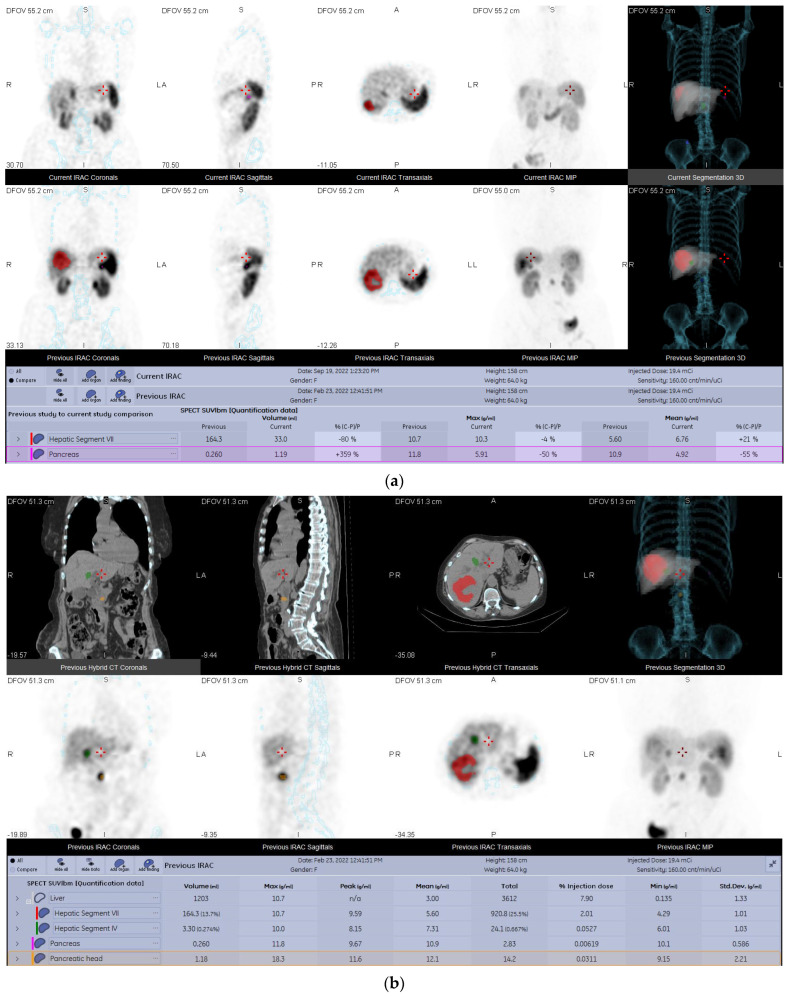

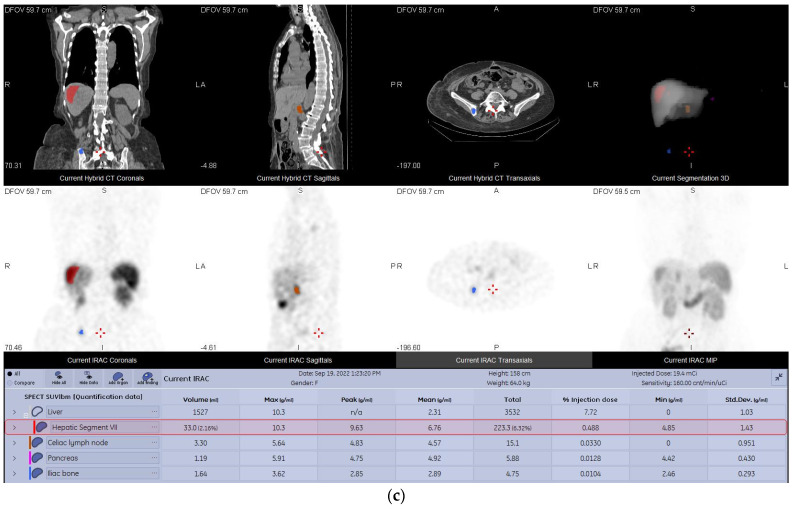
Comparison between a baseline and a follow-up scan of a 69−year−old female patient suffering from pancreatic NET, treated with long acting somatostatin analogues. The comparative analysis between the baseline scan and the follow−up scan (**a**) shows a decrease in uptake both in the primary tumour (the pancreas) and in the liver metastasis. However, when analysing the two lesion segmentation processes separately, compared to the baseline scan (**b**), the follow−up scan (**c**) shows that new lesions developed in the lymph nodes and bones. This patient was finally classified as PD.

Most of the patients (64.29%) in our study presented SD at the end of the study (Figure 3), with only two patients (14.29%) exhibiting PD (Figure 4) and three patients having PR (21.43%). No patients with CR were part of our study.

Out of the four patients that had multiple follow-up scans, one had three follow-up examinations, with a 6-month timeframe for each of them, and presented continuous PD on all of them. The second patient, who also had three follow-up scans, taken 6 months apart each, exhibited SD on the first two scans, but was finally classified as PR on the final examination. The third patient, who also had three follow-up SPECT/CTs, but at irregular intervals (6–11 months), was classified as PD on the first follow up scan, reclassified as PR on the second follow-up examination, and was finally assessed as SD on the last one. The fourth and last patient had two follow-up examinations performed, the first one 12 months after the baseline scan, being assessed as PD, and the second one 16 months after the first scan, where they were classified as SD.

## 4. Discussion

Radiolabeled somatostatin analogues are valuable tools for both the diagnosis and therapy of NETs because of their capacity to bind to SSTR, which are usually overexpressed on the tumoral cellular surface [28]. Although ^68^Ga-DOTA-PET/CT currently represents the gold standard in the diagnosis and monitoring of patients with NETs, SPECT/CT using ^99m^Tc-labelled somatostatin analogues could represent a good alternative in centres that do not have the benefit of PET/CT equipment or ^68^Ge/^68^Ga generators [21].

Quantifying SPECT/CT data has proven useful in other nuclear medicine branches, as with bone tumours or cardiac amyloidosis [29,30,31]. Our study aims to outline its utility in neuroendocrine patient workup, highlighting that, with a precise scanner calibration, it can be a useful tool in monitoring treatment response and disease progression.

The literature regarding quantitative SPECT/CT in NETs is relatively scarce, with few studies published to this point. Reilly et al. [14] conducted a study regarding the characterisation of physiological and neuroendocrine tumour uptake of ^99m^Tc-EDDA/HYNIC-TOC using SPECT/CT SUVs. They observed that SUV_max_ is higher in liver and lymph node metastases (21.8 ± 13.3, respectively 16.3 ± 7.6) than in bone lesions (12.9 ± 7), and certified that SUV_max_ in secondary lesions is notably higher than in normal tissue. Their results are in agreement with ours, as both the SUV_max_ and the SUV_peak_ obtained in our patient group for liver and lymphatic metastases (SUV_max_ 12.44 ± 7.76, respectively 11.98 ± 10.45; SUV_peak_ 10.15 ± 6.79, respectively 8.81 ± 7.99) were much more elevated than the values obtained for bone metastases (SUV_max_ 5.90 ± 3.68; SUV_peak_ 5.55 ± 2.47), and all SUVs for metastatic lesions were higher than the SUVs for normal tissues.

Piwowarska-Bilska et al. [32] researched multiple methods of calculating SUV_max_ and SUV_mean_ values in normal liver and liver metastases in SPECT/CT images with ^99m^Tc-EDDA/HYNIC-TOC. They obtained a mean SUV_max_ value for liver metastases, corrected to lean body mass (lbm), of 27.7 ± 20.3, while the SUV_max_ for normal tissue, calculated in small liver VOI, was 5.7 ± 1.4. Their values were higher than the ones we obtained, but still in agreement with our results, the differences being partly attributed to the dependency of SUV values on scanner calibration and image reconstruction, and also to the different segmentation methods used in their study.

To the best of our knowledge, this is the first study to evaluate the potential utility of quantitative SPECT/CT data in evaluating treatment response and disease progression in patients with neuroendocrine tumours. At present, ^68^Ga-DOTA-PET/CT is considered the standard prognostic tool in NET patients, its data quantification being proven to predict response in PRRT [33,34,35]. A recent study, published by Lee et al. [36], evaluated the utility of SUV measurements on ^68^Ga-DOTATATE PET/CT for predicting progression-free survival on patients diagnosed with gastro-entero-pancreatic NETs treated with SSA. They concluded that SUV_max_ on ^68^Ga-DOTATATE PET/CT could predict the outcome of the treatment independently on the pathologic variables (tumoral grade, ki67 index and mitotic count), as they observed that ^68^Ga-DOTATATE, as a specific ligand for SSTR2, is strongly correlated with a tumours’ SSTR2 expression [36]. The same correlation was found by Miederer et al. [37], who correlated the SUV_max_ of ^68^Ga-DOTATOC PET/CT with the cell membrane-based SSTR2 immunohistochemistry score in 17 patients with NETs, and discovered a strong correlation between the membranous SSTR2 expression and ^68^Ga-DOTATOC uptake. As ^99m^Tc-EDDA/HYNIC-TOC is also known to possess a high affinity for SSTR 2 [19,21], and considering our results, where no correlation between ki67 index and the values of SUV_max_ and SUV_peak_ was found, we can also confirm that SUV values calculated on ^99m^Tc-EDDA/HYNIC-TOC SPECT/CT can work as independent predictors of disease progression and treatment response.

The uptake pattern of ^99m^Tc-EDDA/HYNIC-TOC also correlates with the one observed on ^68^Ga-DOTA-PET/CT. A complex study by Kroiss et al. [38] related that the highest physiological uptake of ^68^Ga-DOTATOC can be observed in spleen (SUV_max_ 32.6 ± 11.8), with lower values for liver (SUV_max_ 12.5 ± 4) and a considerably low uptake for bones (SUV_max_ 1.9 ± 0.8), which is in agreement with the numbers derived from our research: the highest SUV_max_, of 8.01 ± 5.31, for spleen, and a low SUV_max_ in bone tissue of 0.66 ± 0.37. Furthermore, the values of the TL/N ratios we obtained (which correspond to the T/NT ratios on ^68^Ga-DOTA-PET/CT), of 4.20 ± 2.67 for hepatic lesions and of 9.37 ± 2.91 for bone lesions, are close to the results published in their paper, a T/NT ratio for liver of 2.8 ± 1.6 and a T/NT ratio for bones of 10.50 ± 14.2.

In our research, we also analysed the effectiveness of using peak SUV as a diagnostic and prognostic tool. Various studies suggested that SUV_peak_ measured on PET/CT could represent a better alternative to SUV_max_ because it is less affected by image noise [39,40]. SUV_peak_ is defined as the average SUV within a small, fixed size region of interest centred on a high uptake part of the tumour [40]. Regarding the SUV_peak_ measured on SPECT/CT, little information has been documented. The only two studies found, those of Tzortzakakis et al. [41] and Ogura et al. [42] defined SUV_peak_ as the maximum activity concentration in a 1cm^3^ volume of a larger VOI drawn, and described its correlation to the other SUVs measured, SUV_max_ and SUV_mean_. Our research also highlighted the strong positive correlation between the SUV_max_ and SUV_peak_ values (*p* < 0.05), suggesting that a change in SUV_peak_ might also translate into disease progression or treatment response. We chose not to evaluate the SUV_mean_ of the lesions in our studies because of the lack of accuracy in correctly delineating the volume of interest on native CT.

There are, however, some limitations to our study. Firstly, our patient group is comprised of only 14 individuals, which might affect the applicability of our results on a larger scale. Secondly, a standardization of quantitative SPECT/CT has not been well established yet, and although we have performed a rigorous scanner calibration according to the international norms and to the camera vendor’s instructions, further studies need to be performed with regard to this matter. Finally, the variable follow-up period (a range of 4-16 months) might alter the accuracy of patient classification into PERCIST-based groups. Nevertheless, this one last point should have minor implications for our study, as our intention was not to assess the treatment response of our patients, but rather to evaluate the practicability of using quantitative SPECT/CT as a tool in patient monitorization.

## 5. Conclusions

The quantitative analysis of ^99m^TcEDDA/HYNIC-TOC SPECT/CT data in patients with neuroendocrine tumours for evaluating the treatment response could represent an alternative to ^68^Ga-DOTA-peptides PET/CT for patient management, allowing the monitoring of disease evolution and guiding for treatment adjustment. Although the usage of PERCIST-based criteria showed good results, a standardization of the method based on more clinical studies is needed for applicability in clinical practice.

## Figures and Tables

**Table 1:** Patient characteristics.

Age (mean ± SD)	48.36 ± 16.93	(range 18–69)	
**ki67 (mean ± SD)**	7 ± 6.25	(range 2–25%)	
		**Number**	**Percentage**
**Sex**	M	9	64.3%
	F	5	35.7%
**NET localisation**	Pancreas	6	42.9%
	Small bowel	3	21.4%
	Lungs	3	21.4%
	Large bowel	1	7.1%
	Unknown	1	7.1%
**Tumour grade**	NET G1	6	42.9%
	NET G2	7	50%
	NET G3	1	7.1%
**Metastases site**	Liver	11	78.6%
	Lymph nodes	10	71.4%
	Bones	3	21.4%
	Lungs	1	7.1%

Abbreviations: SD—standard deviation; M—male; F—female; NET—neuroendocrine tumour.

**Table 2:** Positivity rate for neuroendocrine markers.

	Positivity Rate (%)
**Immunohistochemical marker**	
CGA	85.7
SYN	92.9
NSE	21.4
CD56	14.3
**Biochemical marker**	
SER	57.1
5-HIAA	14.3
CGA	85.7

Abbreviations: CGA—Chromogranin A; SYN—Synaptophysin; NSE—Neuron Specific Enolase; CD56—Homophilic Binding Protein; SER—Serotonin; 5-HIAA—5-Hydroxyindolacetic acid.

**Table 3:** Tumoral uptake versus healthy tissue uptake of 99mTc-EDDA/HYNIC-TOC in the most frequent sites of the metastases.

Metastatic Site	SUV_max_ of Metastases (g/mL)	SUV_max_ of Healthy Tissue (g/mL)	TL/N Ratio
Liver	12.44 ± 7.76	3.34 ± 0.93	4.20 ± 2.67
Lymph nodes	11.98 ± 10.45	8.01 ± 5.31 (spleen)	1.84 ± 1.53
Bone	5.90 ± 3.68	0.66 ± 0.37	9.37 ± 2.91

Abbreviations: SUV—standardized uptake value; TL/N—target lesion to normal tissue.

**Table 4:** Correlation between SUVmax and SUVpeak of neuroendocrine metastases.

Lesion	SUV_max_ (g/mL)	SUV_peak_ (g/mL)	*r* Correlation Coefficient	*p*
**Liver metastases**	12.44 ± 7.76	10.15 ± 6.79	0.982	0.000
**Lymph node metastases**	11.98 ± 10.45	8.81 ± 7.99	0.980	0.000
**Bone metastases**	5.90 ± 3.68	5.55 ± 2.47	0.920	0.027

Abbreviations: SUV—standardized uptake value; r—a correlation coefficient. *p* < 0.05 was considered as statistically significant.

**Table 5:** Correlation between ki67, SUV_max_ and SUV_peak_ in different tumoral sites.

Lesion—ki67	*r* SUV_max_	*p*	*r* SUV_peak_	*p*
Liver metastases	0.079	0.707	0.127	0.582
Lymph node metastases	−0.036	0.889	−0.012	0.966
Bone metastases	−0. 701	0.506	0.672	0.531
All lesions	0.042	0.820	0.096	0.656

Abbreviations: ki67—proliferation index; SUV—standardized uptake value; r—correlation coefficient.

## Data Availability

Not applicable.
